# Trigeminal Neuralgia Treatment via Piezosurgical Enlargement of the Mental Foramen

**DOI:** 10.3390/life15030382

**Published:** 2025-02-28

**Authors:** Radosław Jadach, Karolina Osypko

**Affiliations:** 1 Private Practice, ul. Eugeniusza Horbaczewskiego 53A, 54-130 Wrocław, Poland; 2 Dental Salon, Oral Surgery Academy, ul. Eugeniusza Horbaczewskiego 53A, 54-130 Wrocław, Poland

**Keywords:** mental nerve, trigeminal neuralgia, facial pain, piezosurgery, surgical treatment, decompression, mental foramen

## Abstract

**Background:** This article and the novel surgical approach described here were inspired by the ideas and observations of the late professors T. Pawela and J. Wnukiewicz. The authors present the medical history and unique surgical treatment of four patients with trigeminal neuralgia, who, despite pharmacological treatment and numerous specialists being involved in the treatment process, continued suffering. Our belief is that the direct cause of the symptoms is a narrow mental foramen, which compresses the mental nerve. It can be easily verified by local anesthesia administration to verify the trigger point, and by analyzing CBCT scans with a special emphasis on the diameter of both mental foramina. **Methods**: Surgical decompression by narrow mental foramen enlargement was conducted with a piezosurgical device. In this procedure, a rectangle of cortical bone is gently and precisely cut around the mental foramen and then into smaller pieces. This technique enables its easy and safe removal. Then, the mental nerve is left loose, uncompressed. **Results**: All four patients reported immediate recovery, their pain attacks stopped, and their quality of life improved significantly. One patient reported temporal hypoesthesia that lasted 5 months post-op. About 2 years post-op, another patient reported rare recurrences of pain, although much less severe than before surgery. **Conclusions**: This type of treatment may be considered when trigeminal neuralgia cannot be classified as classic or as secondary and is unresponsive to pharmacological treatment. A piezosurgical device seems to be the safest option in terms of potential damage to the nerve. Further research should include a larger sample of patients and focus on analyzing the mental foramina diameter of patients with idiopathic trigeminal neuralgia.

## 1. Introduction

Our inspiration to perform research on this topic emerged from the long-forgotten depths of observations made by the Polish professors Tadeusz Pawela and Jan Wnukiewicz, perennial employees of the Maxillo-Facial Surgery department of Wrocław Medical University, at the turn of the 21st century. Pawela and Wnukiewicz noticed that most mental nerve neuralgia reported by their patients was right-sided. What is more, after analyzing the mandibles of the deceased patients, they noted that most right mental foramina had slightly smaller diameters than the left ones; hence, there might be a correlation. No full-scale research was conducted on this topic, although the observation was passed down over time, among surgeons.

Trigeminal neuralgia (TN) is defined by the International Association for the Study of Pain (IASP) as a pain distribution in one or more branches of the fifth cranial nerve, abrupt pain paroxysms, and the provocation of these pain attacks by normally innocuous mechanical stimuli or orofacial movements [1]. Such pain experienced for a longer period of time may result in cognitive impairment, depression, anxiety, social withdrawal, isolation, and even an increased suicide risk [2]. Epidemiological studies show an incidence of 4–27 new cases of trigeminal neuralgia per 100,000 patients annually [3]. It is more commonly reported among women than men (different studies show a ratio of 1.5:1 to 2.3:1 [3]), and the highest incidence occurs between the ages of 50 and 70.

According to the European Academy of Neurology, trigeminal neuralgia can be classified into one of the following categories: primary TN (classic TN and idiopathic TN) and secondary TN [4].

The classical trigeminal neuralgia etiology [5], which is neurovascular compression in the trigeminal root entry zone, is diagnosed by neurologists on the basis of symptoms, as well as by neurosurgeons and MRI analysis to confirm or reject the compression of the nerve by one of the superior cerebellar arteries. In the case of idiopathic TN, which represents about 10% of all TN cases [5], a single clear cause cannot be identified. Secondary TN also arises on the basis of nerve compression, although associated with tumors [6,7] (mostly benign and usually located near the pons [8]) or artery malformations (e.g., multiple sclerosis [8,9]). Secondary TN represents about 15% of all TN cases [8]. Usually, nerve compression leads to the demyelination of nerve fibers (which becomes a trigger for paroxysmal ectopic discharges), although some alterations may occur, including focal demyelination at the entry zone of the trigeminal nerve, the atrophy or hypertrophy of peripheral axons, and damage to Schwann cells, as well as to peripheral myelin [5]. Moreover, trigeminal postherpetic neuralgia (TG-PHN) can be distinguished as a result of varicella zoster virus (VZV) infection [10].

The recommended treatment paths can be divided into conservative and interventional options.

The conservative option focuses on antiepileptic drugs with carbamazepine (200–1200 mg/day) as a gold standard, or oxcarbazepine (300–1800 mg/day) [11,12]. Alternatively, lamotrigine, gabapentin, botulinum toxin type A, pregabalin, baclofen, and phenytoin may be used as a monotherapy or combined with carbamazepine or oxcarbazepine when they alone are insufficient [11,13,14]. New pharmacological alternatives are being considered, such as the active metabolite of oxcarbazepine, eslicarbazepine, and the new Nav1.7 blocker vixotrigine [5].

Pharmacological treatment is the most accessible approach, although for 10% of patients [11], even this option will be insufficient. If the vascular compression of the trigeminal nerve root at the root entry zone (in classic TN) can be confirmed, then a surgical intervention is considered, such as surgical microvascular decompression (MVD), Gamma knife, percutaneous balloon micro compression, glycerol rhizolysis, or percutaneous radiofrequency (RF) treatment of the Gasserian ganglion [5,11,15,16,17].

However, there is a group of patients with diagnosed TN, with typical symptoms, who are non-responsive to drugs and for whom there is no evident cause detectable on MRI. Those cases are classified as idiopathic TN; hence, from here, the treatment plan has no clear path, and patients are usually sent from one doctor to another, looking for help and hope.

Nevertheless, some of those idiopathic TN cases may indeed derive from the compression of myelin on the nerve surface, although the source is not located near the root entry zone, but more peripherally, in the mental foramen. The mental foramen can be roughly compared to a rigid ring of cortical bone surrounding the area of the inferior alveolar nerve (IAN) leaving the mandible body and turning into a mental nerve. With age, this bone ring may shrink and thus start pressing against the surface of the nerve, causing similar results to a vascular compression in classic TN cases.

In Section 3 and Section 4 of this article, the authors present in detail the case of patient B, one of four of our patients whose medical history, symptoms, therapeutic intervention, and results showed similar courses. The differences between the cases were minor, and the authors see no point in describing each case separately.

As such cases have never been described in the medical literature before, this article could provide a relevant introduction to a novel approach to diagnostic strategies and surgical treatment in certain cases of idiopathic TN, where the mental foramen’s diameter is smaller on the symptomatic side in comparison to the asymptomatic side.

## 2. Patient Information

The beginning of the medical history is usually the same—patients start experiencing excruciating pain attacks, sometimes triggered even by a gentle touch, sometimes by a blow of cold wind, or by other “harmless” stimuli. The attacks become more and more frequent and start to preclude normal social functioning. The NSAIDs (nonsteroidal anti-inflammatory drugs) do not bring any relief, since the problem is not caused by any inflammation, but rather by a hyperactivity of the nerves. While searching for any help, patients come to their general dentist hoping to identify its source or for other solutions to alleviate pain. Lacking the odontogenic origin of the symptoms, or after eliminating any potential cause, the dentist refers the patient to a neurologist. Only one of our patients went straight to a neurologist, without a preceding dental appointment.

Based on the reported symptoms, the neurologist diagnoses trigeminal neuralgia, and they are usually the first doctor to prescribe antiepileptic drugs, usually carbamazepine or oxcarbazepine, as they are the first-line recommended medications in many clinical guidelines and reduce the pain suffered by approximately 90% of patients [8]. Unfortunately, the drugs do not improve the situation of our patients, and as MRI contradicts neurovascular compression, the patients are referred again, this time to a maxillo-facial surgeon.

With the lack of a better therapeutic solution, the most common first aid procedure conducted by maxillo-facial surgeons is a block anesthesia performed with two vials (2 × 2 mL) of lignocaine with noradrenaline (Lignocainum 2% c. Noradrenalino 0.00125%) on the mental nerve (if this is the location of a trigger point). While administering the anesthesia, the surgeon purposely tries to enter the mental foramen with a 0.8 mm-diameter needle, as mechanical damage to the nerve may benefit the patient. Then, a second nerve block near the lingula mandibulae is administered. The patient receives such lignocaine blocks once a day for 10 days. The patients report that the problem recurs 2–3 h after the anesthesia, although these 2–3 h are something they could not have achieved otherwise.

As these ten blocks are never sufficient, the eleventh block is a mix of lidocaine and bupivacaine (Bupivacainum Hydrochloricum 0.5%) in a 1:1 ratio. After a few minutes, when the patient is sufficiently anesthetized, 2 mL of 96% ethanol is injected near the mental foramen to chemically destroy the nerve. This treatment usually alleviates a patient’s pain for a few weeks. Nevertheless, after that time, the pain returns. When all chemical means fail, the ultimate decision is the radical surgical treatment and exeresis of the nerve under general anesthesia. Unfortunately, in some cases, the problem recurs after approximately 3 years.

The four patients described in this article came to the clinic before being given lignocaine blocks and exeresis; therefore, this cycle could have potentially been broken before irreversible damage was done. These four cases were included in the study due to: (1) the reported symptoms, (2) the lack of effective solutions provided by a neurologist/neurosurgeon, (3) the CBCT image suggesting the root of the problem being narrow mental foramen, (4) the determination of the patients to try anything that might alleviate their pain, even if the treatment would be considered experimental. These patients represent a small percentage of idiopathic TN cases, as the rest are usually responsive to pharmacological treatment. All cases of classic and secondary TN are treated by a neurologist or a neurosurgeon, and hence they do not fulfill the inclusion criteria of this study. Our patients represent a new, separate group within TN cases with, so far, an imperceptible cause of pain and considerable treatment.

**Figure 1:**
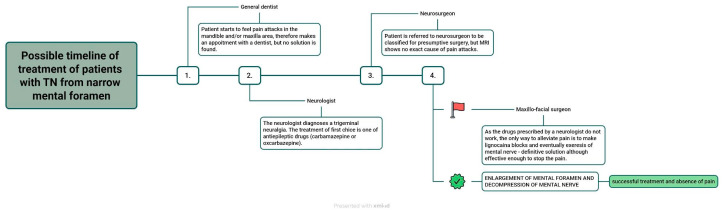
The flowchart of a possible timeline of treatment, containing a neurologist, a dentist, and surgeons.

The timeline of treatment, with two possible endings, is presented in Figure 1.

## 3. Clinical Findings and Diagnostic Assessment

No abnormalities that could be connected to the reported symptoms were detected during the medical examination. Intraorally, the mucosa were healthy, and so were the remaining teeth. The lymph nodes were not enlarged. A decision was made to conduct a CBCT covering the maxilla, the mandible and partially the maxillary sinuses (0.2 mm voxel, FOV—14 cm diameter × 8.5 cm height, GXCB-500 HD, Gendex, Hatfield, PA, USA). The CBCT scans also showed no odontogenic reasons for the reported symptoms. Also, the maxillary sinuses were clear of any radiological signs of inflammation or abnormalities.

As the source of dispersed, neuralgic pain is difficult to localize purely by the affected region, we decided to start giving local block anesthesia at the trigger points and wait a few minutes in between doses to see which localization brought some relief to the patient. This process should begin with the most peripheral regions of the trigeminal nerve, starting from the mental foramen, then the mandibular foramen, suborbital foramen, and supraorbital foramen. About 1 mL of articaine with 1:100,000 adrenaline was used for each point.

**Figure 2:**
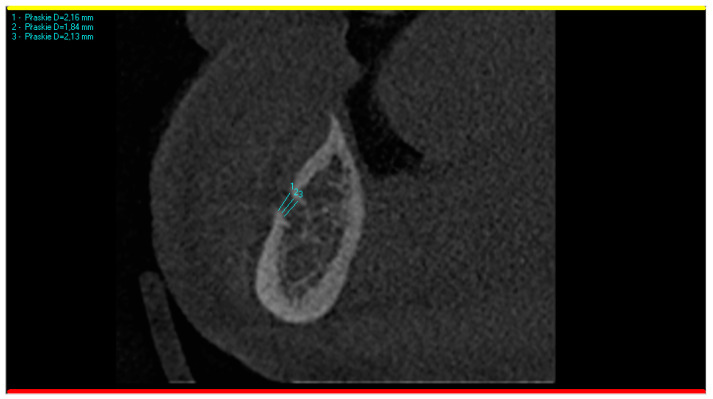
CBCT scan showing the methodology of measuring the mental foramen. Three measurements were taken (results 2.16, 1.84, and 2.13 mm are visible in the upper left corner) and then the mean diameter was calculated (2.04 mm) to obtain the most reliable result.

Fortunately, the patient felt relief just a few minutes after administration of the block anesthesia at the mental foramen. As the approximate cause was related to a mental nerve, the CBCT was analyzed again. Having in mind the hypothesis of professors Pawela and Wnukiewicz, the diameters of the metal foramina were analyzed in comparison with the inferior alveolar canal and with the opposite side. To obtain the most reliable result, each mental foramen was measured three times, and then, the mean vertical diameter was calculated. The measuring was conducted on a CBCT cross-section perpendicular to the panoramic curve, as presented in Figure 2.

The results of these measurements of all four patients are shown in Table 1.

In accordance with the hypothesis of the professors, the neuralgic side had a smaller mental foramen diameter. If the ring of hard cortical bone pressing against the myelin of the surface of the mental nerve was indeed the direct cause of neuralgia, then decompression by the surgical enlarging of the mental foramen could potentially resolve the problem.

Patients were offered the possible surgical solution with emphasis on possible irreversible damage to the nerve, hence loss of sensory and potentially also motoric capabilities, as the V3 branch sometimes contains some motor fibers [18]. All patients agreed on the treatment, as even the perspective of complete loss of feeling in this branch would be beneficial in their condition, meaning the end of pain.

The procedures were conducted in accordance with the Declaration of Helsinki and Medical Code of Ethics. Patients were aware of the possible complications or lack of positive results, as well as of the objective and the process of the proposed intervention. Moreover, patients were informed that they can withdraw from the treatment at any moment.

## 4. Therapeutic Intervention

The patient received local anesthesia of 3 vials of 1.8 mL of articaine 4% with adrenaline 1:100,000. The full-thickness mucoperiosteal flap was elevated, with an incision on the top of the alveolar ridge and with one releasing incision mesially. The flap was carefully elevated around the mental foramen with blunt instruments to prevent puncturing the neurovascular bundle.

When the foramen is localized, the osteotomy around it may begin, with a safety margin of about 5 mm. Moreover, such distance facilitates the procedure, as the proximity of the foramen essentially comprises pure cortical bone of the mandibular body and the mandibular canal. By distancing the osteotomy a few millimeters from the foramen, the piezosurgical tip can cut through to the cancellous bone, hence making it easier to remove bone fragments.

**Figure 3:**
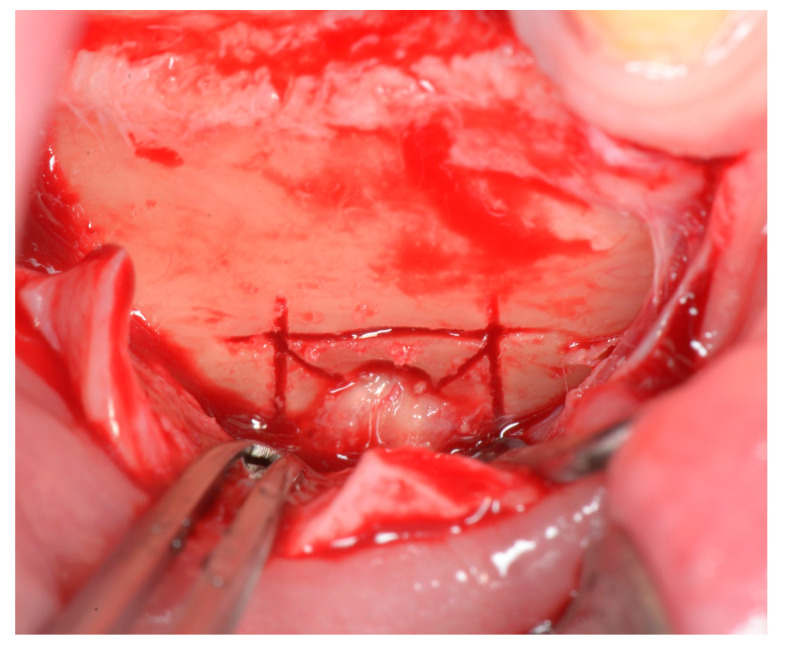
Osteotomy for mental foramen enlargement and mental nerve decompression. Elevating the full-thickness flap unveiled the mental nerve, around which a rectangular osteotomy could be performed. Cross sections on the edges ensure definitive cuts. And finally, diagonal cuts help with sectioning the bone ring into smaller pieces and removing them without damaging the nerve.

Initial osteotomy has a rectangular shape around the foramen, with at least a 5 mm distance from the bundle. The so-called “cross sections” at the edges are important, and they surpass the rectangular lines by a millimeter or two (Figure 3). This results in cross-shaped cuts on the edges of the osteotomy window and ensures that there is no trabecula remaining, which potentially could prevent the clean separation of the bone fragment and compromise the procedure.

Now, when the bony rectangular area around the foramen is disconnected from the surrounding cortical bone, a new challenge arises—how does one remove it?

**Figure 4:**
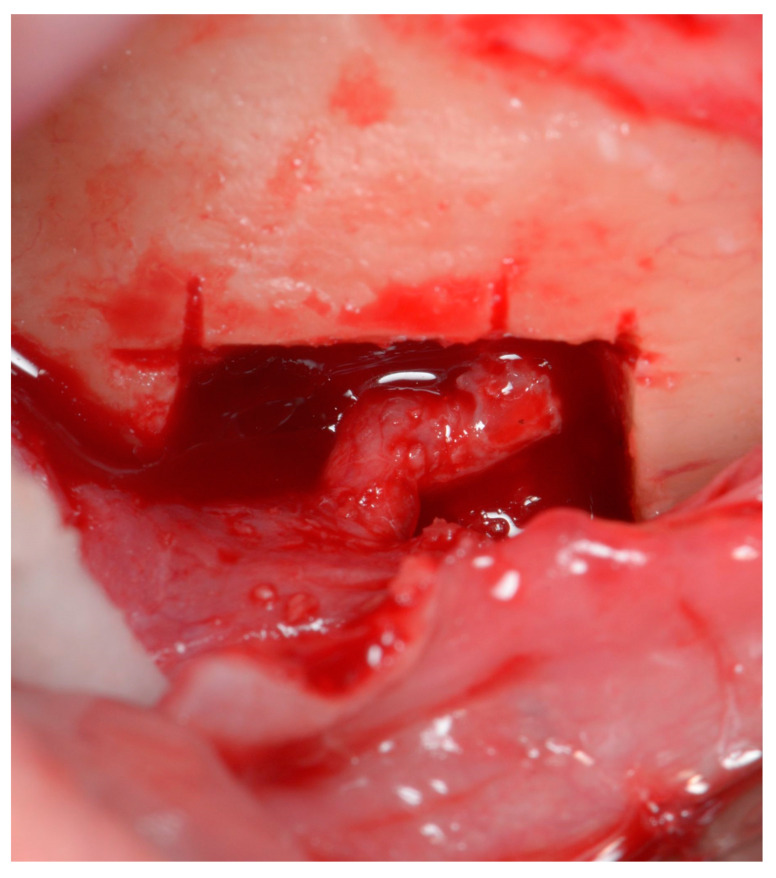
Mental foramen after enlargement and decompression of the nerve. The bundle is left loose; therefore, it has a curved shape.

The second part of osteotomy creates diagonal lines from the corners of the rectangular area to the foramen, with special care not to damage the neurovascular bundle. Although piezosurgical tips are considered to cause very little damage to soft tissues, when used with excessive force, they may still result in irreversible injury [19]. The diagonal cuts enable the sectioning of the rectangular piece of the bone ring into four smaller separate pieces. If they are mobile, they can be easily removed. In case they are still fixed, due to the trabeculae of the cancellous bone, the pieces should be gently luxated with a rigid root elevator (or another rigid surgical tool). Using elastic tools deprives the operator of feeling how the bone behaves under applied force; hence it could potentially jeopardize the procedure, and it is therefore not recommended. When all bone pieces are mobile, they should be gently removed one by one, leaving the mental nerve loose (Figure 4).

**Figure 5:**
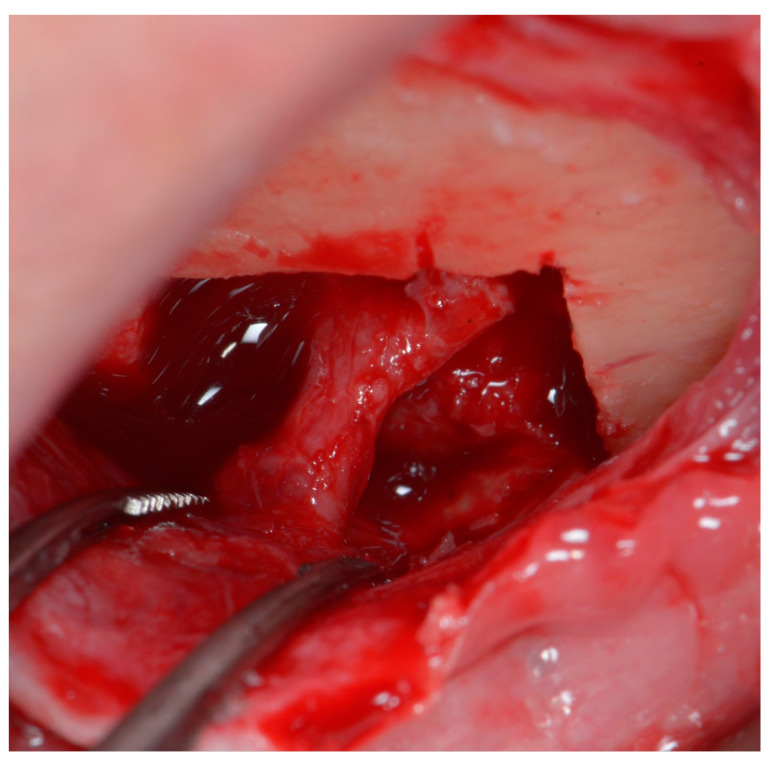
The nerve straightens with a gentle pull of the flap. This signifies that the nerve is loose and there is no compression on its surface.

The operator may see that the bundle is indeed loose, as it straightens under gentle manipulation and pulling the flap (Figure 5), confirming the releasing effect of the procedure.

## 5. Follow-Up and Outcomes

All four patients felt immediate relief after the procedure. The pain attacks stopped, and the patients regained their comfort of living. The results of the Visual Analog Scale (VAS) [20], which is a scale of perceived pain, ranging from 0 (no pain) to 10 (agonizing pain), dropped from 8.75 to 0.50 after the procedure.

In three out of four cases, there was no post-op hypoesthesia. One patient (patient C) reported subtle numbness for 5 months.

In the follow-up appointment 12 months post-op, all patients were free of neuralgic symptoms. About 2 years post-op, one patient (patient A) reported the seldom recurrence of pain attacks, although much lighter than before, and usually only during colder months. The reason for this might be the healing process of the operated area and the possible regrowing of the cortical bone around the mental foramen. This patient was invited for follow-up CBCT to verify this hypothesis, although due to their advanced age (over 80 years old) and other health issues, the appointment had to be postponed.

## 6. Discussion

The following types of surgical interventions are most frequent in the case of detecting neurovascular compression with morphological changes of the nerve: microvascular decompression, balloon compression, and glycerol rhizolysis. Microvascular decompression consists of craniectomy and approaching the cerebellopontine angle where the trigeminal nerve and the compressing vessel are located. Then, either the vessel is transposed (and fixed with Teflon and glue) or the Teflon piece interposes the nerve and the vessel [21]. Balloon compression is a technique whereby, under the neuronavigation system, the needle is inserted through the skin into the oval foramen. After reaching the trigeminal ganglion, the balloon is inflated with contrast medium (to confirm its position) and the ganglion is compressed for two minutes. Next, the balloon and the needle are removed [21]. In the case of glycerol rhizolysis, the oval foramen is reached with a needle, similarly as in the balloon compression technique. Then, the patient’s head is properly set, and 0.8 mL of glycerol is injected with the needle to chemically affect the nerve. The patient’s head is fixed with a custom-made pillow in one position for 1 h to ensure its activity is concentrated in the Meckel’s cave [21]. These interventions may result in numerous complications, including postoperative meningitis, hypoesthesia, hearing impairment, ischemic stroke, or even death directly connected to the surgery [21].

In cases like the ones described in this article, when the cause of pain is related to the nerve compression, although not near the trigeminal root entry zone but on the “periphery”, which is the mental foramen, most of the above-mentioned complications are simply excluded. The only one reported by our patients was hypoesthesia, although, as mentioned before, after years of pain, the sensation of numbness is perceived as a blessing by those patients.

Nevertheless, any complication, no matter how small, should be avoided if possible. This idea led to the implementation of a piezosurgical device in our cases.

Piezosurgery, implemented in the context of dentistry by Tomaso Vercellotti [22], is a significantly safer tool for osteotomy around crucial soft tissue structures, such as nerves, than traditional rotary instruments and surgical burs [23,24,25]. In dentistry, it is widely used in bone harvesting, ridge splitting, sinus lifting, apicoectomy, implant-site osteotomy, or the lateralization of the inferior alveolar nerve and more complex maxillo-facial surgeries [25,26]. To be even more precise, among the procedures related to the inferior alveolar nerve are its lateralization [19] and decompression after the accidental overfilling of the mandibular canal with endodontic sealer [27].

Lateralization is an alternative procedure to bone augmentation, when there is not enough bone height above the mandibular canal and the patient wants implants [28]. The whole idea is to open the access through the osteotomy to the mandibular canal from the lateral side, gently retrieve the nerve, and place it outside of the mandible body, so it can run between the periosteum and the cortical bone. In that way, the whole mandible body’s height can be utilized for implants.

The decompression of the mandibular canal from the endodontic sealer is an emergency procedure [29]. If during endodontic treatment some sealer was accidently pushed through the apex and neurovascular bundle to the mandible canal, it may cause irreversible damage, e.g., hypoesthesia. The only solution is quick intervention and rinsing the canal with physiological saline solution, thus it requires creating surgical access through osteotomy.

In both these procedures, a piezosurgical device enables cutting through the hard, mineralized cortical bone, and leaving the IAN itself [28] untouched or maybe minimally scratched. Certainly, there is always a risk of irreversible damage when enlarging the mental foramen, although when compared to the potential risks during and after neurosurgical operation, it is undeniably of a different scale, especially given that nerve exeresis was for years (or still is) a widely practiced method of treatment. The sole use of a piezosurgical device is not a novelty in surgery, although this study reveals its possible new application in TN treatment, if the patient qualifies for the mental foramen enlargement procedure.

The main limitation of this technique is its narrow application, that is, only those cases wherein the mental foramen of the affected side is indeed of a smaller diameter than the one of the asymptomatic side. If during a neurologist’s examination, a neurovascular compression (classic TN), a tumor, or any other secondary TN is detected, then naturally the cause of the patient’s symptoms lies elsewhere. Although, if no obvious reason is found with MRI, the clinician’s attention should be focused on the mental foramen, as its enlargement is fairly safe and may be the final remedy for the patient’s problem.

The presumed pathophysiological process of the reported symptoms lies within the superficial compression of the mental nerve by the cortical bone of the mental foramen. The remodeling of the mandible may progress due to the extraction of teeth, compression by partial or complete dentures, or simply by age, and hence, may lead to the repositioning of the cortical bone and the “shrinkage” of the mental foramen. This situation later results in the focal demyelination of the mental nerve, which becomes the trigger of paroxysmal ectopic discharges. As the surgical technique described in this study not only leaves the nerve loose, but at the same time almost eliminates the reported symptoms, the above-mentioned hypothesis is supported. To confirm this, a histopathological examination should be conducted, although that would require a resection of the nerve and cause irreversible damage to the patients.

After analyzing the available literature, no similar procedure has ever been described. A reasonably similar procedure of infraorbital canal (IOC) decompression in patients with TN symptoms was described by Han et al. [30], which confirms the reasoning for a similar procedure on the mental foramen. In the above-mentioned study, the procedure was conducted under general anesthesia, with a piezosurgical device and an endoscope. In the case of the mental nerve decompression described here, the important advantage is local anesthesia and a relatively simpler surgical procedure, which can be performed by any experienced oral or maxillo-facial surgeon.

Although the work of doctors Pawela and Wnukiewicz on the comparison of mental foramen diameters was never finished, this topic should be thoroughly studied today, especially in the context of a potential new neuralgia treatment.

Among available studies, the one by Mallahi et al. [31] analyzed CBCT scans of 355 healthy patients. One of the parameters here was the vertical diameter and width of the mental foramina (mm), with division by presence on the left and right side, as well as the sex and age of the patient (above or below 45 years). A statistical difference was found between the mental foramen widths on the left (4.15 ± 1.31) and right (4.37 ± 1.30) sides. Moreover, the mental foramen’s vertical diameter was different in men (4.07 ± 1.71) and women (3.73 ± 1.48). Finally, on the right side, the mental foramen’s vertical diameter was smaller in younger patients (≤45 years; 3.95 ± 1.46) and bigger in older patients (>45 years; 4.34 ± 1.73).

Only the conclusion here regarding sex differences is in accordance with our observations. On the other hand, that research was conducted among unaffected patients; therefore, future studies should focus on analyzing CBCT scans of patients with diagnosed neuralgia, especially with idiopathic TN, when no neurovascular compression is found on the MRI. A close collaboration between dentists, oral surgeons, maxillo-facial surgeons, neurologists and neurosurgeons may lead to new approaches and better solutions, and give hope to the affected patients.

## 7. Conclusions

The enlargement of the mental foramen may be a promising and fairly safe treatment method for patients with diagnosed trigeminal neuralgia who are not suffering from a classic or secondary TN confirmed by MRI. Before implementing this technique, the diameters of the mental foramina should be compared via CBCT. The piezosurgical device, thanks to its properties, can be considered the most suitable choice for preparing a rectangular osteotomy around the foramen, with lower risk of damaging the IAN. Nevertheless, patients must be informed about possible hypoesthesia after the procedure. After the procedure, all our patients reported instant, long-term relief, although due to a small study sample, this topic requires more extended research in the future.

This article was written according to CARE guidelines.

## Figures and Tables

**Table 1:** Measurements of the diameter of the mental foramen on both sides of the four patients who underwent the enlargement of the mental foramen (decompression of the mental nerve).

Patient	Sex	Age	Neuralgic Side (Causal)	Diameter of Mental Foramen at the Neuralgic Side (mm)	Diameter of Mental Foramen at the Oppoite, Indolent Side (mm)
A	F	82	right	2.04	2.55
B	M	72	right	2.34	3.11
C	F	69	right	2.20	2.63
D	F	59	left	1.80	2.45

## Data Availability

Data are contained within the article.

## Abbreviations

CBCT	Cone beam-computed tomography
IAN	Inferior alveolar nerve
TN	Trigeminal neuralgia
VAS	Visual Analog Scale
